# Predictors of Seizure Freedom in Pediatric Low-Grade Gliomas

**DOI:** 10.7759/cureus.31915

**Published:** 2022-11-26

**Authors:** Hailey C Budnick, Shawyon Baygani, Teresa Easwaran, Alexander Vortmeyer, Andrew Jea, Virendra Desai, Jeffrey Raskin

**Affiliations:** 1 Neurological Surgery, Indiana University School of Medicine, Indianapolis, USA; 2 Radiation Oncology, University of Minnesota School of Medicine, Minneapolis, USA; 3 Pathology and Laboratory Medicine, Indiana University Health, Indianapolis, USA; 4 Neurological Surgery, Oklahoma University Health Sciences Center, Oklahoma City, USA; 5 Pediatric Neurological Surgery, Ann & Robert H. Lurie Children’s Hospital of Chicago, Chicago, USA

**Keywords:** seizures, epilepsy, engel outcome, pediatric low grade glioma, pediatric epilepsy surgery

## Abstract

Objective: Pediatric low-grade gliomas (LGGs) are found in approximately one to three percent of patients with childhood epilepsy. Epilepsy in these patients is often medically refractory and therefore represents a unique cohort with significant morbidity from concomitant pathology. Similar studies in adult patients with low-grade gliomas have identified predictors of seizure freedom including gross-total resection, preoperative seizure control on antiepileptic medication and duration of seizures of less than one year. This study aims to identify similar predictors of seizure freedom in operatively managed pediatric LGGs.

Methods: A retrospective chart review was performed for patients diagnosed with World Health Organization (WHO) Grade I and II gliomas in patients ≤18 years old at a single institution (Indiana University School of Medicine at Riley Hospital for Children in Indianapolis, IN) from 2007-2017. Infratentorial and purely intraventricular lesions were excluded. WHO classification and histologic diagnosis were based on surgical pathology. Tumor grade, location, laterality, seizure status at presentation, and AED requirements pre- and post-operatively were recorded. Chi-squared analyses for independence were performed controlling for age at presentation, resection extent, seizure type, and Engel Class for seizure freedom post-operatively.

Results: Forty-two patients met the inclusion criteria. Preoperative seizures were observed in 23 patients (55%). Presentation with preoperative seizures was highly associated with continued seizure burden post-operatively, independent of the extent of surgical resection. Supratentorial location and the administration of prophylactic pre- and post-operative AEDs were associated with Engel Class I seizure freedom. Temporal location was not significantly associated with medically refractory epilepsy compared with extra-temporal locations.

Conclusions: In our cohort of pediatric LGGs, we find that patients that did not initially present with seizures and those who were treated with prophylactic pre- and post-operative AEDs, were more likely to achieve Engel Class I seizure freedom post-operatively. Tumors located in the temporal location were not significantly associated with a higher seizure burden than other supratentorial, extra-temporal tumors. Neither extent of resection nor electrocorticography-guided resection correlated with improved seizure freedom outcomes during glioma resection.

## Introduction

Adults and children with low-grade gliomas (LGGs) often present with seizures which can lead to medically refractory epilepsy [1-4]. LGGs, including glial and glioneuronal tumors, are found in roughly one to three percent of patients with childhood epilepsy; such seizures often present significant morbidity, often more so than other tumor-related symptoms [5-7].

Slow-growing tumors such as dysembryoplastic neuroepithelial tumors (DNETs) and gangliogliomas are more likely than high-grade tumors to be epileptogenic in children [8,9]. The grade of neoplasm is often more life-limiting than the epileptic sequelae in both the pediatric and adult populations; however, when tumor-related epilepsy remains uncontrolled, it significantly contributes to patient morbidity and negatively impacts the quality of life [10,11].

Approximately 70% of all patients with LGGs endure a seizure during the disease process; 30%-50% of them have seizures on presentation, while approximately 9%-30% of patients have seizures as their only presenting symptom [3]. A recent multisite retrospective study found that 24% of pediatric patients with supratentorial LGGs also had seizures on presentation, and that these patients could be stratified into medically refractory, more than two and less than two seizures [12]. They report a very low rate of post-operative new-onset epilepsy and a significant decrease in epilepsy in these pediatric patients with LGGs. In adult patients presenting with LGGs and epilepsy, predictors of seizure freedom have been identified including gross-total resection, preoperative seizure control on antiepileptic medication and duration of seizures of less than one year [10,13]. Tumor location also influences the risk for epilepsy, with those localized in the frontal, temporal and parietal lobes being more commonly associated with seizures than those in the occipital lobes [14,15]. This study aimed to identify predictors of seizure freedom of LGGs among the pediatric population given their significant differences and diversity in histopathology, as well as the duration of disease burden both of the tumor itself and the potential for a secondary seizure disorder.

## Materials and methods

A retrospective chart review was performed of all patients at Riley Hospital for Children, with new diagnosis of low-grade glioma between January 1, 2000 and December 31, 2017. Inclusion criteria consisted of surgical resection or biopsy, age ≤18 years at diagnosis, follow-up for at least one year, and pathologic diagnosis of low-grade glioma (grade I or II) as determined by a board-certified pathologist. Posterior fossa and purely intraventricular lesions were excluded due to their typical non-epileptigenic nature. Patients who had lifelong or pre-existing epilepsy were also excluded if it was clear that the epilepsy was present before the tumor. 

Comparative oncologic data included date of diagnosis, pathology, anatomical location, the extent of surgical resection, adjuvant radio- or chemotherapy, recurrence rates and progression-free and overall survivals (PFS and OS). The extent of resection was defined as biopsy, subtotal resection leaving behind any residual amount, and gross total resection. Residual or recurrence was identified by a board-certified neuroradiologist using MRI with and without gadolinium contrast.

Seizure data included status at presentation, whether the patient had experienced a seizure at any point in time prior to presentation, anti-epileptic medication use pre- and post-operatively (usually Levetiracetam but Phenytoin was also used in some patients), and Engel class outcome. 

Statistical analysis

Chi-square analyses with and without Yates correction were performed on calculated pooled rates of seizure freedom (Engel Class I) versus continued seizure burden (Engel Classes II-IV). Values reported include Yates correction if significance was changed, otherwise reported without correction. Initial between-group comparisons were performed using the Pearson chi-squared test with Yates correction as all variables were categorical. Odds ratios with a 95% confidence interval were performed to analyze variables found to be significant (p<0.05) following chi-squared analysis with ratios reported in regard to seizure freedom with a 95% confidence interval. Statistical and survival analyses were conducted using Prism 8 software.

## Results

A total of 42 patients met inclusion criteria with an average age of 9.76 +/- 4.9 years and 27 patients (64%) achieved Engel class I postoperatively and 15 patients (35%) Engel Classes II-IV. The mean follow-up was 58.7 +/- 39.5 months with a median PFS of 30.1 months. Complete demographic information is shown in Table 1. 

**Table 1:** Demographic information AED - Anti-epileptic drugs; GTR - Gross total resection; STR - Subtotal resection

Age at Diagnosis (years ± SEM)	9.76 ± 4.9
Gender (# F (%))	24 (57)
Tumor Pathology	n (%)
Astroblastoma	1 (2)
Ependymoma	1 (2)
Fibrillary low-grade astrocytoma	2 (5)
Ganglioglioma	2 (5)
Juvenile pilocytic astrocytoma	22 (52)
Oligoastroytoma	6 (14)
Oligodendroglioma	2 (5)
Pilomyxoid astrocytoma	1 (2)
Pleomorphic xanthoastrocytoma	5 (12)
Tumor Grade	
I	23 (55)
II	19 (45)
Tumor Laterality	
Left	21 (50)
Right	21 (50)
Tumor Location	
Frontal	10 (24)
Frontoparietal	1 (2)
Frontotemporal	1 (2)
Hypothalamus	1 (2)
Insula	1 (2)
Optic Nerve	2 (5)
Parietal	5 (12)
Retro-orbital	1 (2)
Temporal	15 (36)
Thalamus	4 (10)
Midbrain/ Thalamus	1 (2)
Seizures at Presentation	
Yes	23 (55)
No	18 (43)
Unknown	1 (2)
Pre-operative AEDs	
Yes	30 (71)
No	12 (29)
Post-operative AEDs	
Yes	28 (65)
No	14 (35)
Surgery	
Biopsy	2 (5)
GTR	25 (60)
STR	15 (35)
Chemotherapy	
Yes	22 (52)
No	20 (48)
Radiation	
Yes	23 (56)
No	19 (44)
Engel Class	
1	27 (64)
2	11 (26)
3	3 (7)
4	1 (2)
Recurrence	
Yes	18 (43)
No	24 (57)
Hydrocephalus	
Yes	9 (21)
No	33 (79)

The majority of tumors were astrocytic (70%) with a nearly even split between grade I, n=22 (51%) and grade II, n=21 (49%). Sixteen (38%) were located in the temporal lobe with the remainder extra-temporal (62%). Most patients presented with seizures pre-operatively and 30 patients (71%) presented on at least one anti-epileptic drug (AED), with three patients (7%) presenting with greater than one year of seizure duration. Of the patients who were Engel 2-4, 15 patients (94%) presented with seizures and all 16 were on at least one AED.

Seizure status at presentation (p<0.001) and pre- (p<0.01) as well as post-operative AED use (p<0.001), and recurrence (p<0.01) had a statistically significant association with Engel outcome (Table 2). However, when looking at the causal relationship, the most significant variables tested were pre-operative and post-operative AED use (Table 3). Pathology, grade, location, laterality, the extent of resection, and adjuvant chemo- or radiotherapy were not significantly associated with seizure freedom. Details of demographics and comparison of variables by pooled Engel class outcome are shown in Tables 1, 2.

**Table 2:** Comparison of variables by pooled Engel class outcome AED - Anti-epileptic drugs; ECoG - Electrocorticography

Seizure Freedom	Engel 1 (n=27)	Engel 2-4 (n=15)	P-value
Age at Diagnosis (years ± SEM)	10.19± 5.26	9.00 ± 4.19	P = 0.75
Gender (#F (%))	15 (56)	9 (60)	P = 0.78
Seizure at Presentation (n(%))	10 (38)	13 (87)	P < 0.01
Seizure Duration (pre) >1yr (n(%))	2 (17)	1 (8)	P = 0.49
Tumor Pathology (n(%))			P = 0.34
Astroblastoma	1 (4)	0	
Ependymoma	0	1 (7)	
Fibrillary low-grade astrocytoma	1 (4)	1 (7)	
Ganglioglioma	1 (4)	1 (7)	
Juvenile pilocytic astrocytoma	17 (63)	5 (33)	
Oligoastroytoma	3 (11)	3 (20)	
Oligodendroglioma	2 (7)	0	
Pilomyxoid astrocytoma	0	1 (7)	
Pleomorphic xanthoastrocytoma	2 (7)	3 (20)	
Tumor Grade			P = 0.15
I	17 (63)	6 (40)	
II	10 (37)	9 (60)	
Tumor Laterality			P = 0.63
Left	15 (56)	6 (40)	
Midline	0	0	
Right	12 (44)	9 (60)	
Tumor Location			P = 0.26
3^rd^ Ventricle	0	0	
Frontal	4 (15)	6 (38)	
Frontoparietal	0	1 (7)	
Frontotemporal	1 (4)	0	
Hypothalamus	0	1 (7)	
Insula	1 (4)	0	
Optic Nerve	2 (7)	0	
Parietal	4 (15)	1 (7)	
Retro-orbital	1 (4)	0	
Temporal	9 (33)	6 (40)	
Thalamus	5 (9)	0	
Pre-operative AEDs			P <0.01
Yes	15 (56)	15 (100)	
No	12 (44)	0	
Post-operative AEDs			P < 0.001
Yes	13 (48)	15 (100)	
No	14 (52)	0	
Surgery			P = 0.80
Biopsy	1 (4)	1 (7)	
Gross Total Resection	17 (63)	8 (53)	
Subtotal Resection	9 (33)	6 (40)	
Chemotherapy			P = 0.17
Yes	12 (44)	10 (67)	
No	15 (56)	5 (33)	
Radiation			P = 0.25
Yes	13 (48)	10 (67)	
No	14 (52)	5 (33)	
Intraoperative ECoG			P = 0.57
Yes	5 (42)	4 (31)	
No	7 (58)	9 (69)	
Recurrence			P < 0.01
Yes	7 (26)	11 (73)	
No	20 (74)	4 (27)	
Hydrocephalus			P = 0.30
Yes	7 (26)	2 (13)	
No	20 (74)	13 (87)	

**Table 3:** Chi-square analyses and odds ratios for factors implicated in seizure freedom GTR - Gross total resection; AED - Anti-epileptic drugs; ECoG - Electrocorticography

	$\chi$^2^	p-value	OR
GTR	0.45	0.799	
Seizure at presentation	8.98	0.003	0.10 (0.020 - 0.519)
Extratemporal (supratentorial) vs. temporal	0.04	0.850	
Pre-op AEDs administered	9.33	0.002	0.04 (0.002 – 0.740)
Post-op AEDs administered	11.67	<0.001	0.03 (0.002 – 0.553)
Hydrocephalus present	0.91	0.341	
Use of ECoG intraoperatively	0.32	0.571	
Recurrence	8.85	0.003	0.12 (0.030 – 0.533)
Duration of seizures ≥1 year	0.476	0.490	

## Discussion

Achieving seizure freedom for patients with LGGs is one of the primary treatment goals in controlling tumor-associated morbidity and mortality in afflicted patients [16-18]. This goal is of higher importance in pediatric populations in whom the neurodevelopmental impacts of seizures have been shown to lead to lifelong deficits in academic, social, and emotional competencies [19-22]. In our retrospective review of LGG cases at Riley Hospital for Children, we analyzed the factors associated with seizure freedom following operative care. In adults, the most important predictor for seizure freedom in LGGs has been shown to be gross-total resection; however, in our study, we did not observe this same effect [10,13]. Additionally, we did not see the significant impact of other adult predictors for seizure freedom including seizure type or duration of seizures.

The effect of electrocorticography-guided resection on epilepsy outcomes is an active area of research. Studies in similar adult patients demonstrate statistically and clinically significant improvements in post-operative seizure freedom [23]; however, this has not been demonstrated in the pediatric population [24]. Despite routine use at our institution, we found that electrocorticography-guided resection was also not associated with higher rates of seizure freedom. This could potentially be due to electrocorticography not playing a large role in intra-operative surgical guidance, but this point deserves more attention.

The mainstay of LGG treatment remains surgical resection in both adult and pediatric patients [25]. Previous adult studies suggest the extent of resection plays an important role in Engel's outcome, with some suggesting 80%-90% resection as the threshold for seizure freedom [13,25,26]. Importantly in our study, resection was stratified as biopsy only, subtotal and gross-total, while the extent of subtotal resection was not further classified.

Pre- and post-operative AED use was associated with increased seizure burden, as expected, implying that those patients with decreased burden had a lower AED requirement. In addition, patients with a history of seizures at presentation were less likely to achieve seizure freedom. These results mirror those of a previous multi-site pediatric study [27].

The models in which seizure freedom is assessed have varied between studies. Pediatric population studies report post-operative seizure occurrence prior to one week [27,28] and one month [12] was a negative prognostic sign for seizure freedom. Similar adult cohort studies have used 6-12 months post-operatively to define Engel's outcome [25,29]. The desirability of this patient perspective may in some way be lost in children as another layer of subjectivity is added by a parent’s appraisal of their child’s disability. Epilepsy is stigmatizing and socially isolating, potentially leading to parental underreporting of seizure burden as parents eagerly wish for their child to regain normal functioning [30]. In our study, seizure freedom was classified according to the patient’s status at the last clinical follow-up, which ranged from 11 to 148 months with a mean of 58.7 +/- 39.5. Engel class rates of 64% recorded at five years are longer than typically reported and compare favorably to the literature [26,28].

A primary limitation of our study was the patient sample size which limited the power of our study. For example, in a study of 253 patients by Chang and colleagues, improvements in seizure freedom by GTR were found to have an effect size of approximately 0.3, as calculated by Cramer’s V. For this effect size to be observed, a sample size of 46 would be necessary, slightly larger than in our study. Additionally, the retrospective nature of the study limits the validity of our conclusions to the extent of our data accuracy.

## Conclusions

In our cohort of pediatric LGGs, we find that patients that did not initially present with seizures and those who were treated with pre- and post-operative AEDs were more likely to achieve Engel Class I seizure freedom post-operatively. Tumors located in the temporal lobe were not significantly associated with a higher seizure burden than other supratentorial, extra-temporal tumors. Neither extent of resection nor electrocorticography-guided resection correlated with improved seizure freedom outcomes during glioma resection.
